# Xiaoyao San ameliorates maternal inflammation-induced neurobehavioral deficits by modulating the microbiota-gut-brain axis in offspring

**DOI:** 10.3389/fphar.2025.1563496

**Published:** 2025-05-19

**Authors:** Chunqiao Lin, Jiushuang Zhu, Lu Zhang, Lijie Shi, Zhuoting Zhong, Xiuwen Xia, Weijun Ding, Youjun Yang

**Affiliations:** ^1^ School of Basic Medical Sciences, Chengdu University of Traditional Chinese Medicine, Chengdu, China; ^2^ Sichuan College of Traditional Chinese Medicine, Mianyang, China

**Keywords:** Xiaoyao san, neurodevelopmental disorders, maternal immune activation (MIA), gut microbiota, amino acid transporter

## Abstract

**Background:**

XiaoYao San (XYS), a classical Traditional Chinese Medicine (TCM), has demonstrated efficacy in alleviating stress-related neuropsychiatric disorders. However, its therapeutic potential against maternal immune activation (MIA)-induced neurobehavioral impairments remains unexplored. This study aims to investigate the neuroprotective effects of XYS on MIA-related behavioral dysfunctions and elucidate its underlying mechanisms.

**Results:**

Using a poly (I:C)-induced MIA mouse model, we demonstrated that XYS effectively ameliorates autism spectrum disorder (ASD) related behavioral phenotypes. Mechanistic investigations revealed that XYS exerts its therapeutic effects through: (1) Attenuation of core behavioral deficits including enhanced social interaction and reduced repetitive behaviors; (2) Downregulation of intestinal amino acid transporters; (3) Restoration of cerebral glutamate-GABA balance via modulation of glutamine pathway; (4) Structural remodeling of gut microbiota with specific enrichment of *Bacteroides* spp. Notably, *B. uniformis* was identified as a key microbial mediator capable of recapitulating XYS-mediated neurophysiological improvements through metabolic regulation.

**Conclusion:**

This study elucidates XYS as a multi-target therapeutic agent that coordinately modulates gut microbial ecosystems, amino acid homeostasis, and neurotransmitter homeostasis. The findings provide novel insights into the gut-brain axis mechanisms of TCM formulations, offering a scientific foundation for developing microbiota-based intervention strategies for neurodevelopmental disorders.

## 1 Introduction

Neurodevelopmental disorders (NDDs) cover a range of conditions that impact cognitive, behavioral, and social skills (Parenti et al., 2020). They typically appear during childhood and persist into adulthood. These disorders, such as attention deficit hyperactivity disorder (ADHD), autism spectrum disorder (ASD), and schizophrenia, are increasingly recognized as significant global health challenges (Gidziela et al., 2023). While the exact causes of NDDs remain unknown, a growing body of research suggests that early life factors, including maternal immune activation (MIA), play a critical role in shaping neurodevelopmental trajectories (Knuesel et al., 2014; Han et al., 2021). MIA, induced by infections or inflammatory processes during pregnancy, is associated with an increased risk of ASD in offspring (Knuesel et al., 2014; Estes and McAllister, 2016; Careaga et al., 2017). Activating the maternal immune system produces cytokines and other immune mediators that can cross the placental barrier and influence fetal brain development, potentially resulting in long-term behavioral impairments (Choi et al., 2016; Rudolph et al., 2018). The Poly (I:C)-induced MIA model in rodents has been widely used to elucidate how maternal immune challenges can impact offspring neurodevelopment, providing insights into the pathophysiology of ASD (Zuckerman et al., 2003; Kim et al., 2017; Kentner et al., 2019).

The gut microbiome in the gastrointestinal tract is a complex ecosystem of microorganisms crucial in influencing host brain functions and behaviors (Cryan and Dinan, 2012; Morais et al., 2021). Recent research has shown a bidirectional communication network between the brain and the gut microbiota, potentially impacting neurodevelopment and behavior (Morais et al., 2021). Dysregulation of the gut microbiome has been implicated in ASD (Sharon et al., 2019; Yap et al., 2021; Morton et al., 2023; Ahrens et al., 2024; Su et al., 2024), suggesting that gut microbiome dysbiosis may cause ASD. However, some studies also show no direct link between gut microbiome and ASD (Yap et al., 2021). Previous studies have revealed a significant link between MIA and the alterations of gut microbiota in offspring (Hsiao et al., 2013; Tartaglione et al., 2022b), demonstrating that MIA not only induces gastrointestinal abnormalities and behavioral traits akin to ASD in mice but also leads to a distinct dysbiosis characterized by changes in microbial composition. Importantly, studies have shown that these MIA-induced gut microbial imbalances can be mitigated by treatment with *Bacteroides fragilis* (Hsiao et al., 2013), suggesting a potential therapeutic approach targeting the *Bacteroides* to alleviate ASD-related symptoms (Hsiao et al., 2013; Buffington et al., 2016; Mazzone et al., 2024).

Traditional Chinese medicine (TCM) offers a rich source of potential treatments for various conditions, including mood and behavioral disorders. Xiaoyao San (XYS), a classical Chinese herbal formula, is known for its ability to soothe the liver and regulate the spleen (Wang Y. T. et al., 2023). It has been used for centuries to treat various conditions related to emotional distress and digestive discomfort (Xie et al., 2023). We and other groups have reported that certain herbal compounds in XYS interact with the gut microbiota, leading to changes in microbial composition and metabolic activities that could potentially improve anxiety- and depression-related behaviors (Yang et al., 2022; Hao et al., 2024; He et al., 2024). It's important to note that while these findings are promising, the research in this area is still in the early stages, and more comprehensive clinical trials are needed to confirm the efficacy and mechanisms of action of XYS in modulating the gut microbiota and improving neurobehavioral health. Integrating traditional herbal medicine with modern microbiome research offers a fascinating frontier for developing novel therapeutic strategies for neurological and psychiatric conditions. The potential of XYS to ameliorate MIA-induced behavioral abnormalities and its underlying mechanisms warrant further exploration, particularly in the context of the gut-brain axis.

Our study aims to investigate the therapeutic potential of XYS in a Poly (I:C)-induced MIA model, focusing on its effects on behavioral abnormalities and its capacity to modulate the gut microbiome. By examining the interplay between XYS treatment, the gut microbiome, and neurobehavioral outcomes, we aim to contribute to understanding the microbiome-gut-brain connection in the context of NDDs and explore the potential of TCM as a therapeutic strategy for these disorders.

## 2 Materials and methods

### 2.1 Experimental animals

The specific pathogen-free (SPF) C57BL/6J mice (8 weeks old) were obtained from Sibeifu Biotechnology Co., Ltd. (Beijing, China). The mice were housed in a specific pathogen-free facility (25°C, humidity range of 40%–50%) and maintained in a 12 h light: dark cycle. The mice were allowed access to food and water freely. Mating occurs at night, and female mice are examined for seminal plugs the next morning, which is recorded as embryonic day 0.5 (E0.5). On E12.5, the pregnant mice received an intraperitoneal injection of Poly (I:C) (20 mg/kg, Sigma Aldrich, P9582) (Kim et al., 2017; Shin Yim et al., 2017). After Poly (I:C) injection, the dams were put back into their home cage and left undisturbed until giving birth. The pups were weaned on postnatal day 21 (P21). After weaning, three to five offspring with the same sex were group-housed. All procedures involving the animals complied with the Guidelines for the Care and Use of Laboratory Animals established by the Institution Animal Care and Use Committee at Chengdu University of Traditional Chinese Medicine (CDUTCM.No20221001c20240130).

### 2.2 XYS treatment

XYS combines eight Chinese herbs; the detailed information of the composition of XYS is shown in Table 1. The XYS used in the study was purchased from the Affiliated Hospital of Chengdu University of TCM. Different concentrations of XYS were prepared: 0.975 g/kg (XYS-L), 1.95 g/kg (XYS-M), and 3.9 g/kg (XYS-H). Mice in the XYS treatment group were gavaged with XYS once a day for 4 weeks. The reported doses refer to the crude weight of the raw herbal materials prior to extraction, not the actual administered dose of the extract. The decoction was prepared via water extraction (traditional method) with a standardized 15% yield, resulting in a water extraction dose of 0.14625 g/kg (XYS-L), 0.2925 g/kg (XYS-M), and 0.585 g/kg (XYS-H). Control mice were given equivalent volumes (200 μL) of saline daily. Qualitative analysis of XYS was performed using a QExactive TMHF/Q ExactiveTM HF-X mass spectrometer by Novogene Co., Ltd. (Beijing, China).

**TABLE 1:** The composition of XYS.

Herb Name	Latin Name	Chinese Name	Weight (g)	Medical part
*Radix Bupleuri*	*Bupleurum chinensis DC.*	Chai Hu	100	Root
*Radix Angelicae sinensis*	*Angelica sinensis (Oliv.) Diels*	Dang Gui	100	Root
*Poria*	*Poria cocos (Schw.) Wolf*	Fu Ling	100	Sclerotium
*White Paeony Root*	*Peaonia lactiflora Pall*	Bai Shao	100	Root
*Rhizoma Atractylodis Macrocephalae*	*Artactylodes macrocepha Koidz*	Bai Zhu	100	Root
*Rhizoma Zingiberis Recens*	*Zingiber officinale Rosc*	Sheng Jiang (wei)	20	Root
*Herba Menthae*	*Mentha haplocalyx Briq*	Bo He	20	
*Radix Glycyrrhizae*	*Glycyrrhiza uralensis Fisch*	Gan Cao (zhi)	80	Root

### 2.3 Three-chamber test

The three-chamber social interaction test involves a setup with a neutral middle chamber and two side chambers holding either a stimulus animal or an object. Mice are first adapted to the environment, and then their interactions with an unfamiliar mouse versus an inanimate object (sociability test) and a new, unfamiliar mouse (social novelty test) are observed in different phases. Each phase lasts 10 min, and the mouse’s time spent in each chamber is recorded to assess social interest and memory. Data is captured using XinRuan’s SuperMaze/VisuTrack software (Shanghai, China), and the apparatus is cleaned between trials to eliminate odor interference.

### 2.4 Marble burying test

The marble burying test is commonly used to assess repetitive, stereotyped behavior in animals, particularly rodents like mice (Thomas et al., 2009; Angoa-Pérez et al., 2013). In this test, mice are placed into testing arenas with specific dimensions (40 cm × 20 cm × 30 cm) and bedding depth (3 cm). The arenas contain 20 glass marbles arranged in four rows of five marbles, each equidistant from one another. During a 15-min exploration, the mice interact with the marbles, and their behavior is observed. After this period, the mice are carefully removed from the testing cages, and the number of marbles buried by the mice is recorded. The marble burying index is used to quantify the behavior observed, with a defined scale: a value of one is assigned to marbles covered by bedding more than 50%, a value of 0.5 for marbles covered by bedding less than 50%, and a value of 0 for marbles that are not covered at all. This index quantitatively measures the mice’s repetitive and burying behavior.

### 2.5 Forced swimming test

The forced swim test is a widely respected method for evaluating depression-like behavior in mice, making it an essential tool in behavioral research. In this experiment, mice are placed in a transparent Plexiglas cylinder filled with water, maintained at a temperature of 24 °C and a depth of 15 cm. Their behavior is recorded for 6 min with a camera positioned overhead, allowing for precise observation. Following the recording, researchers meticulously analyze the duration of immobile floating during the final 5 min using advanced tracking software (XinRuan, XR-XJ117), providing valuable insights into their emotional status.

### 2.6 Tail suspension test

The tail suspension test is frequently employed to assess rodent depressive behavior. In this test, mice are suspended by their tails onto an apparatus, and the duration of their immobility is recorded. Immobility is considered to be an indicator of depressive-like behavior, and the total experimental recording time lasted 6 min. During the last 4 min of the test, the mice’s immobility was tracked using XinRuan tracking software (XR-XJ117), and the resulting data were meticulously analyzed by an independent observer.

### 2.7 Elevated plus maze (EPM) test

The EPM (XinRuan, XR-XZ201, Shanghai) test is an essential behavioral assay for accurately assessing rodents' exploratory activity and anxiety-like behavior, particularly in mice and rats. Firstly, the test mice acclimate to the testing environment and the maze for 10 min to ensure reliable and unbiased results. It is then carefully placed in the maze’s center, facing a closed arm. This strategic positioning creates an unbiased starting point for the exploration. During the subsequent 5-min testing phase, the number of entries into each section of the maze and the time spent in the open and closed arms is meticulously recorded using advanced video tracking software SuperMaze/VisuTrack (XinRuan, Shanghai, China). The time spent in the open arms and the frequency of entries are direct indicators of anxiety-like behavior. At the same time, the measurement of movement speed provides critical insight into the animal’s motor abilities. The apparatus is thoroughly cleaned after use, eliminating any residual odors that could impact future tests. This attention to detail ensures the reliability and validity of the findings, making the EPM a cornerstone of anxiety research.

### 2.8 Open-field test (OFT)

The OFT was conducted following established procedures. Each test mouse was individually placed in a 30 × 30 cm open-field arena, and their movements were recorded for 5 min. The recorded videos were analyzed using the offline video tracking software SuperMaze/VisuTrack (XinRuan, Shanghai, China). The time spent in the central zone (15 × 15 cm) and the number of entries into this area were recorded.

### 2.9 Enzyme-linked immunosorbent assays (ELISA)

We conducted a thorough analysis of maternal serum cytokine concentrations at 3 and 24 h following the injection of either Poly (I:C) or PBS, which is crucial for understanding immune responses during pregnancy. Blood samples were meticulously obtained from pregnant dams through a precise cardiac puncture technique. After anesthetizing the animals with sodium pentobarbitone (50 mg/kg, i.p.), we ensured they were prone to optimal access. By targeting the site of the most muscular cardiac beats on the left side with a syringe, we successfully collected approximately 0.5–0.6 mL of blood from each mouse. Following this, we centrifugated at 3,000 rpm for 10 min, and the supernatant was preserved at −80 °C for reliable analysis. The quantification of cytokine levels was carried out using a mouse IL-6 and IL-17α ELISA kit (Elabscience) in strict accordance with the manufacturer’s guidelines, utilizing a Varioskan LUX microplate reader (Thermo Fisher) for accurate measurements. This comprehensive approach enhances the credibility of our findings and contributes valuable insights into maternal immune responses.

### 2.10 Quantitative real-time polymerase chain reaction (qPCR) analysis

To extract total RNA from the medial prefrontal cortex (mPFC), we utilized TRIzol Reagent from Invitrogen, meticulously following the manufacturer’s protocol. This precision allowed us to proceed with cDNA synthesis, where we rigorously assessed its quantity and purity using established methodologies. By quantifying the absorbance ratios at 260/280 nm, we ensured the integrity and high quality of the extracted RNA; a ratio within the 1.8 to 2.1 range confirms that our RNA is both undegraded and well-purified. For our quantitative PCR (qPCR) analyses, we implemented the cutting-edge StepOnePlus Real-Time PCR System from Applied Biosystems alongside the SYBR Green PCR Master Mix, also from Applied Biosystems. Our qPCR protocol included an initial step at 95°C for 30 s, followed by 40 cycles of 95°C for 5 s, 60°C for 30 s, and 72°C for 1 min. Data analysis was conducted using the 2^−ΔΔCt^ method (Livak and Schmittgen, 2001), with *Gapdh* as the reference housekeeping gene. Detailed information on specific primer sequences utilized in this study is available in Supplementary Table S1.

### 2.11 Immunohistochemistry

The test mice were anesthetized using sodium pentobarbital (50 mg/kg, i.p.) before undergoing intracardiac perfusion with saline at 37°C, followed by ice-cold paraformaldehyde (PFA, 4% in 0.1 M PBS) perfusion. Then, the brains were post-fixed overnight at 4 °C in 4% PFA. These brains were subsequently immersed in 10%, 20%, and 30% sucrose solutions (in 0.1 M PB), with each solution incubated overnight to ensure thorough infiltration. The brain is coronally sliced into 30 μm thick with Leica cryostat CM1950. Brain slices were washed three times with PBST (0.3% Triton X-100) and blocked with 5% donkey serum in PBST for 2 h at room temperature for the immunocytochemistry analysis analysis. Following this blocking step, the slices were incubated overnight at 4 °C with the primary antibodies. After washing with PBST three times, the slices were incubated with secondary antibodies for 2 h at room temperature. After these steps, the slices were washed three times with PBST and carefully mounted on slides. Images were captured using a Leica TCS SP8 confocal microscope. The quantitative analysis of GFAP^+^, Iba1^+^, PV^+^, and c-Fos^+^ cells in bilateral mPFC mice was conducted using ImageJ software (https://imagej.nih.gov/ij/). Detailed information about the primary and secondary antibodies used in this research can be found in Supplementary Tables S2, 3.

### 2.12 16s rRNA sequencing

The DNA of fecal samples was extracted using the DNeasy PowerSoil Kit (Qiagen). The 16S rRNA sequencing was conducted by Novogene Co., Ltd. (Beijing, China) using the Illumina MiSeq sequencing platform. VSEARCH software determines Operational taxonomic units (OTUs) at a 97% similarity threshold. Then, the chosen reads underwent detailed annotation and a thorough BLAST search against the SILVA database. To gain insight into microbial diversity in the fecal samples, we calculated α-diversity by focusing on the count of observed species. Finally, we computed the weighted and unweighted UniFrac principal coordinate analysis using QIIME software.

### 2.13 *Untargeted metabolomics* (LC-MS/MS)

Serum and fecal samples were collected and quickly cryopreserved in liquid nitrogen and stored at −80 °C. The untargeted metabolomics analysis was carried out by Novogene Co., Ltd. (Beijing, China) by using a Vanquish UHPLC system and an Orbitrap QExactive HF-X mass spectrometer, and the samples were divided into negative polarity modes in UHPLC-MS/MS analysis. We used Compound Discoverer 3.1 to process the raw data from UHPLC-MS/MS systematically. Furthermore, Compound Discoverer matched peaks with mzCloud, mzVault, and MassList databases. Metabolite annotation was performed using the trusted Kyoto Encyclopedia of Genes and Genomes (KEGG) database.

### 2.14 RNA sequencing (RNA-seq)

Total RNA from the mPFC of mice was extracted by using TRIzol (Invitrogen) according to the manufacturer’s protocols. After RNA extraction, we conducted first-strand cDNA synthesis and library preparation with the NEBNext Ultra Directional RNA Library Prep Kit for Illumina (New England Biolabs) in strict accordance with the instructions provided. mRNA sequencing was conducted by using the NovaSeq 6000 platform (Illumina). Then, HISAT2 (version 2.0.5) and StringTie (version 1.3.4) pipelines were employed for precise transcript quantification and mapping. Differential expression gene (DEG) analysis was determined by using DESeq2 (version 1.24.0) with a rigorous significance threshold of adjusted P ≤ 0.05 and |log_2_(CF)| ≥ 0. Finally, the identified DEG underwent Gene Ontology (GO) term enrichment analysis.

### 2.15 *Bacteroides* uniformis (B. uniformis) treatment

*B. uniformis* (ATCC 8492, BiobW Biotechnology Co., Ltd., Beijing) was grown anaerobically in Gifu Anaerobic Broth at 37°C. For *B. uniformis* administration, the bacteria were centrifuged and suspended in PBS to achieve a 5 × 10^8^ CFU/mL concentration. Then, the *B. uniformis* suspension was administered orally to the mice (0.1 mL/mouse) by gavage for 4 weeks.

### 2.16 Amino acid analysis

Blood and brain tissue samples were immediately frozen in liquid nitrogen upon collection. We used commercially available assay kits to analyze amino acids, following the manufacturers' precise instructions. The glutamine (Gln) levels in both blood and brain tissue were quantified using the Gln Colorimetric Assay Kit (cat#: K556-100, BioVision). To assess glutamate (Glu) concentrations in blood and brain samples, we employed the reliable Glu ELISA Kit from Renjiebio (cat#: RJ17145). Furthermore, we evaluated γ-amino butyric acid (GABA)levels in the mPFC using the mouse GABA ELISA Kit from Biorbyt (cat#: Orb782385).

### 2.17 Statistical analysis

The statistical analysis for this study was carried out using Prism software from GraphPad. We carefully evaluated the data to ensure it followed a normal distribution and presented the results in the figures as mean values with their corresponding standard error of the mean (SEM). The variable “n” indicates the number of independent biological replicates in each figure. We did not exclude any samples or animals from the analyses to maintain the integrity of the data. To determine the significance of differences between the two treatment groups, we employed a two-tailed, unpaired Student’s t-test. For situations involving more than two groups with a single variable, we utilized one-way ANOVA followed by a Bonferroni *post hoc* test to assess where differences lay between the groups. In cases where two variables were under consideration, we implemented a two-way ANOVA with a Bonferroni *post hoc* test to provide a comprehensive analysis. Statistical significance was defined as a *P* ≤ 0.05. Detailed statistical information for each figure can be accessed in Supplementary Table S4.

## 3 Results

### 3.1 XYS rescues autism-like behavior in MIA offspring

**FIGURE 1:**
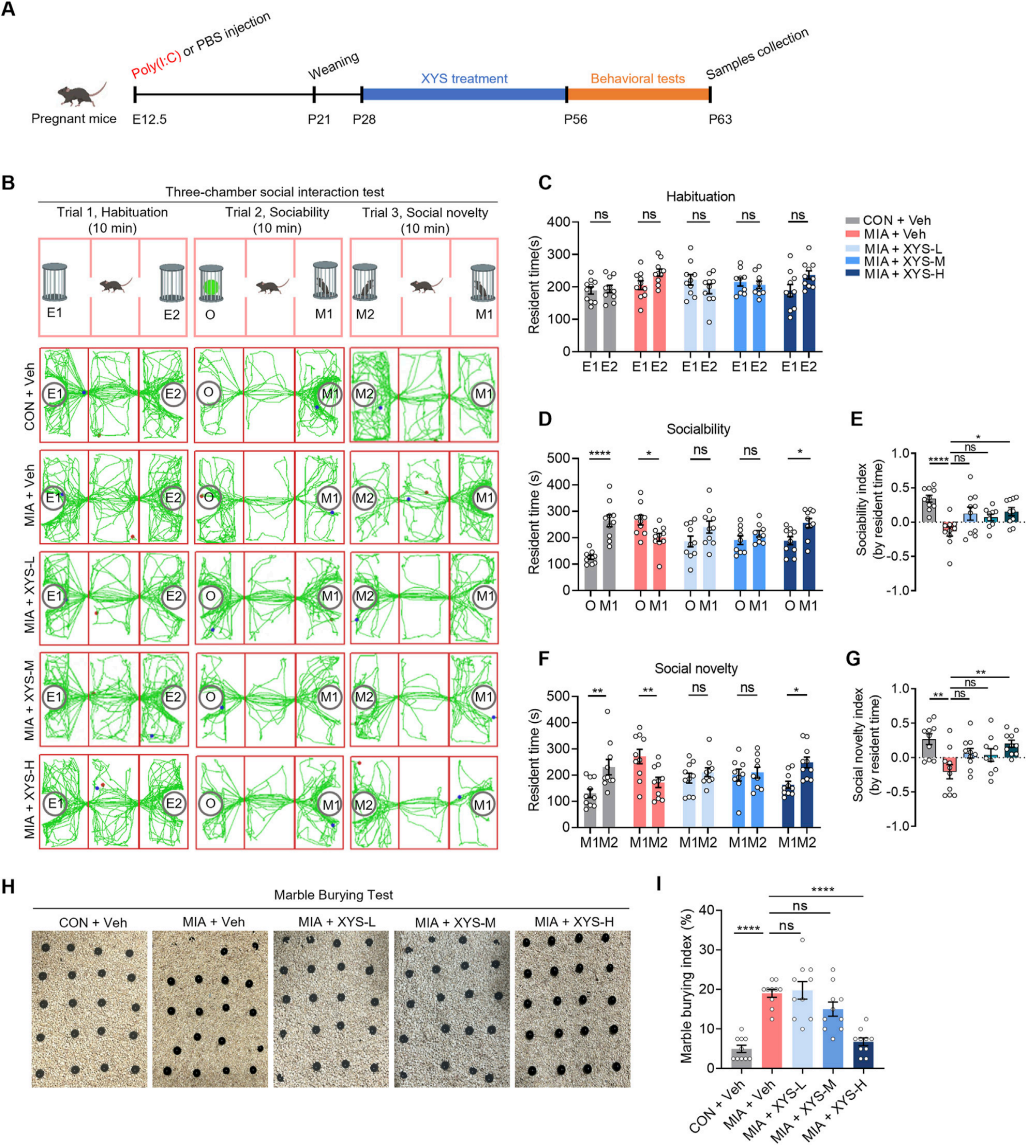
XYS rescues ASD-like behaviors in MIA offspring. **(A)** The experimental timeline XYS treatment and behavioral tests. **(B)** Schematic of the three-chamber social interaction test and representative traces of test mice in this test. **(C)** The resident time in chambers of the test mice in the trial of habituation. **(D)** The resident time in chambers of the test mice in the trial of sociability. **(E)** The sociability index of the test mice, sociability index = (time in M1 – time in O)/(time in M1 + time in O). **(F)** The resident time in chambers of the test mice in the trial of social novelty. **(G)** The social novelty index of the test mice, social novelty index = (time in M2 – time in M1)/(time in M1 + time in M1). **(H)** Representative images of marble-burying test for the mice. **(I)** Quantification of the marble-burying index of test mice (n = 9–10 mice from different dams for each group). Graphs are mean ± SEM. Statistical details are provided in Supplementary Table S4.

HPLC-MS/MS were used to identify the compounds from XYS. Several bioactive compounds, including Galloylpaeoniflorin, Liquiritin, Demethoxycurcumin, Ferulic acid, Saikosaponin A, and Glycyrrhizic acid, were detected in the XYS (Supplementary Figure S1A, B). To investigate whether XYS administration can ameliorate autism-like behavior in MIA offspring, we performed the XYS treatment on the MIA mice from P28 to P56 (Figure 1A). We observed an increase in maternal plasma IL-6 levels at 3 h and IL-17α levels at 24 h after the injection of Poly (I:C), confirming the induction of inflammation in dams (Supplementary Figure S2A, B). While the bodyweight of the offsprings were not significantly changed by MIA at adult (Supplementary Figure S2C). As previously reported (Tartaglione et al., 2022a; Griego et al., 2025), MIA offspring exhibited impaired social behaviors in the three-chamber social interaction test (Figures 1B–G). Importantly, other studies have shown that there is no affectation on social behavior in the offspring (Lan et al., 2023). Notably, high-dose XYS (XYS-H) treatment robustly rescued MIA offspring’s sociability and social novelty deficits compared to (Figures 1B–G). Low-dose and middle-dose XYS (XYS-L and XYS-M) treatment slightly improves the sociability and social novelty in MIA offspring but not significantly (Figures 1B–G). Additionally, the administration of XYS-H suppressed the marble-burying behavior in MIA offspring (Figures 1H,I), which suggests that XYS can relieve the repetitive behavioral phenotypes. Unlike in autism-like behavior, however, XYS treatment did not affect the anxiety- and depression-related behaviors in MIA offspring (Supplementary Figures S3A–L). These data suggest that the XYS-H treatment may only apply to a specific set of core behavioral traits associated with autism in this particular mouse model of neurodevelopmental disorder.

### 3.2 XYS alters the gut microbiome in MIA offspring

**FIGURE 2:**
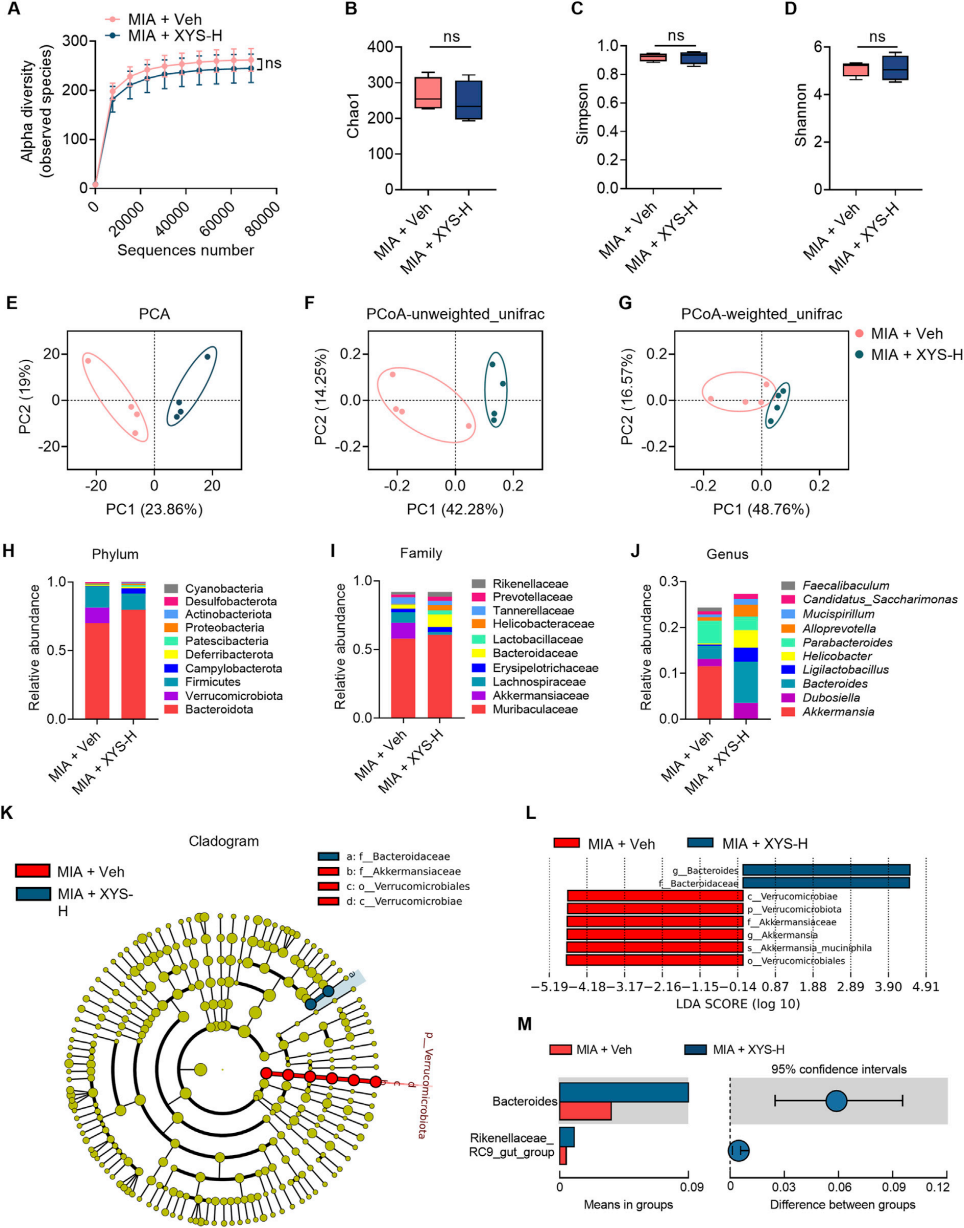
XYS increases the relative abundance of *Bacteroides* in MIA offspring. **(A–D)** α diversity of fecal 16S rRNA sequencing data from MIA + Veh and MIA + XYS-H mice (n = 4 mice from different dams for each group). **(E)** PCA of fecal 16S rRNA sequencing data from MIA + Veh and MIA + XYS-H mice (n = 4 mice from different dams for each group). **(F, G)** Unweighted and weighted UniFrac PCoA of fecal 16S rRNA sequencing data from MIA + Veh and MIA + XYS-H mice (n = 4 mice from different dams for each group). **(H–J)** Relative abundances of the top 10 bacterial phylum, family, and genus from fecal 16S rRNA sequencing data (n = 4 mice from different dams for each group). **(K)** Cladogram showing the phylogenetic relationships of bacterial taxa was revealed by LEfSe (n = 4 mice from different dams for each group). **(L)** Bar chart showing the log-transformed LDA scores of bacterial taxa identified by LEfSe analysis (n = 4 mice from different dams for each group). **(M)** XYS increased the levels of *Bacteroides* in MIA offspring. Graphs are mean ± SEM. Statistical details are provided in Supplementary Table S4.

Research has identified abnormalities in the gut microbiota of individuals with ASD (Sharon et al., 2019; Ahrens et al., 2024; Su et al., 2024). Additionally, prior research has demonstrated that MIA causes dysbiosis in gut microbiota (Hsiao et al., 2013; Tartaglione et al., 2022b), primarily due to changes in specific OTUs within the bacterial classes Clostridia and Bacteroidia (Hsiao et al., 2013). Therefore, to investigate whether XYS improves autism-like behavior in MIA mice by modulating the gut microbiome, We investigated the fecal bacterial composition by performing 16S rRNA gene sequencing on samples collected from vehicle- and XYS-treated MIA offspring at P63. The α diversity of gut microbiota was not significantly changed by XYS-H treatment, as indicated by the observed species, Chao1, Simpson, and Shannon indices (Figures 2A–D). Then, principal component analysis (PCA) (Figure 2E) and principal coordinate analysis (PCoA) (Figures 2F,G) were utilized to evaluate the phylogenetic similarity of microbial communities. The findings demonstrated a significant impact of XYS-H treatment on the gut microbiota of MIA offspring, highlighting notable differences in gut microbiota composition between vehicle-treated and XYS-H-treated MIA offspring (Figures 2E–G). The impact of XYS on altering the gut microbiota is further evident through taxa summary at the phylum, family, and genus levels (Figures 2H–J). Cladogram generated by LEfSe analysis showed the abundant clades of f_Bacteroidaceae, f_Akkermansiaceae, o_Verrucomicrobiales, and c_Verrucomicrobiae are different between vehicle- and XYS-H-treated MIA mice (Figures 2K, L). Furthermore, XYS administration increased the relative abundance of *Bacteroides* in MIA mice (Figure 2M). Overall, XYS-H treatment alters the gut microbiome and primarily increases specific OTUs of the bacterial genus of *Bacteroides* in MIA offspring.

### 3.3 B. uniformis supplementation alleviates ASD-like behaviors in MIA offspring

**FIGURE 3:**
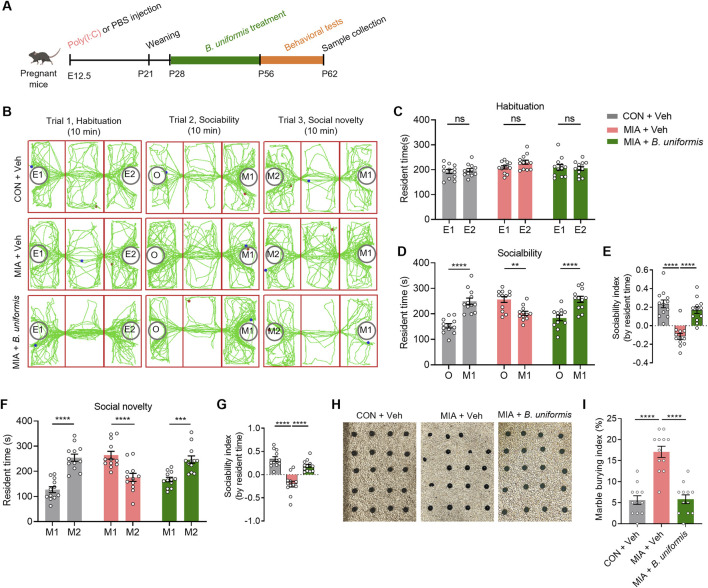
*Bacteroides uniformis* ameliorates the ASD-like behaviors in MIA offspring. **(A)** The experimental timeline *Bacteroides uniformis* treatment and behavioral tests. **(B)** Representative traces of test mice in three-chamber social interaction test. **(C)** The resident time in chambers of the test mice in the trial of habituation. **(D)** The resident time in chambers of the test mice in the trial of sociability. **(E)** The sociability index of the test mice, sociability index = (time in M1 – time in O)/(time in M1 + time in O). **(F)** The resident time in chambers of the test mice in the trial of social novelty. **(G)** The social novelty index of the test mice, social novelty index = (time in M2 – time in M1)/(time in M1 + time in M1). **(H)** Representative images of marble-burying test. **(I)** Quantification of the marble-burying index of the test mice (n = 12 mice from different dams for each group). Graphs are mean ± SEM. Statistical details are provided in Supplementary Table S4.

Gut microbiota is essential in developing, maintaining, and repairing the intestinal epithelium (Hsiao et al., 2013; Sommer and Backhed, 2013). Our results showed that XYS administration increased the relative abundance of the genus of *Bacteroides* in MIA mice. To determine whether XYS could improve ASD-like phenotypes and decrease the intestinal amino acid transport in MIA offspring by targeting the *Bacteroides*, MIA mice were gavaged with *Bacteroides uniformis* (*B. uniformis*) once every day for 4 weeks (P28-P56) (Figure 3A). *B. uniformis* has demonstrated the ability to mitigate behaviors associated with ASD and restore the E/I ratio in the brain (Yu et al., 2022). This effect is achieved by reducing intestinal amino acid transport and lowering serum Gln levels in the Chd8^+/−^ ASD mouse model (Yu et al., 2022). Interestingly, our results showed that treatment with *B. uniformis* improved the social interaction deficits observed in MIA mice (Figures 3B–G). Additionally, the supplementation of *B. uniformis* reduced the increased marble-burying behavior observed in the offspring of MIA (Figures 3H,I). These data suggest that *B. uniformis* can relieve the behavioral phenotypes associated with MIA.

### 3.4 XYS decreased intestinal amino acid transporter levels and altered the Glu/GABA ratio in the serum and brain of MIA offspring

**FIGURE 4:**
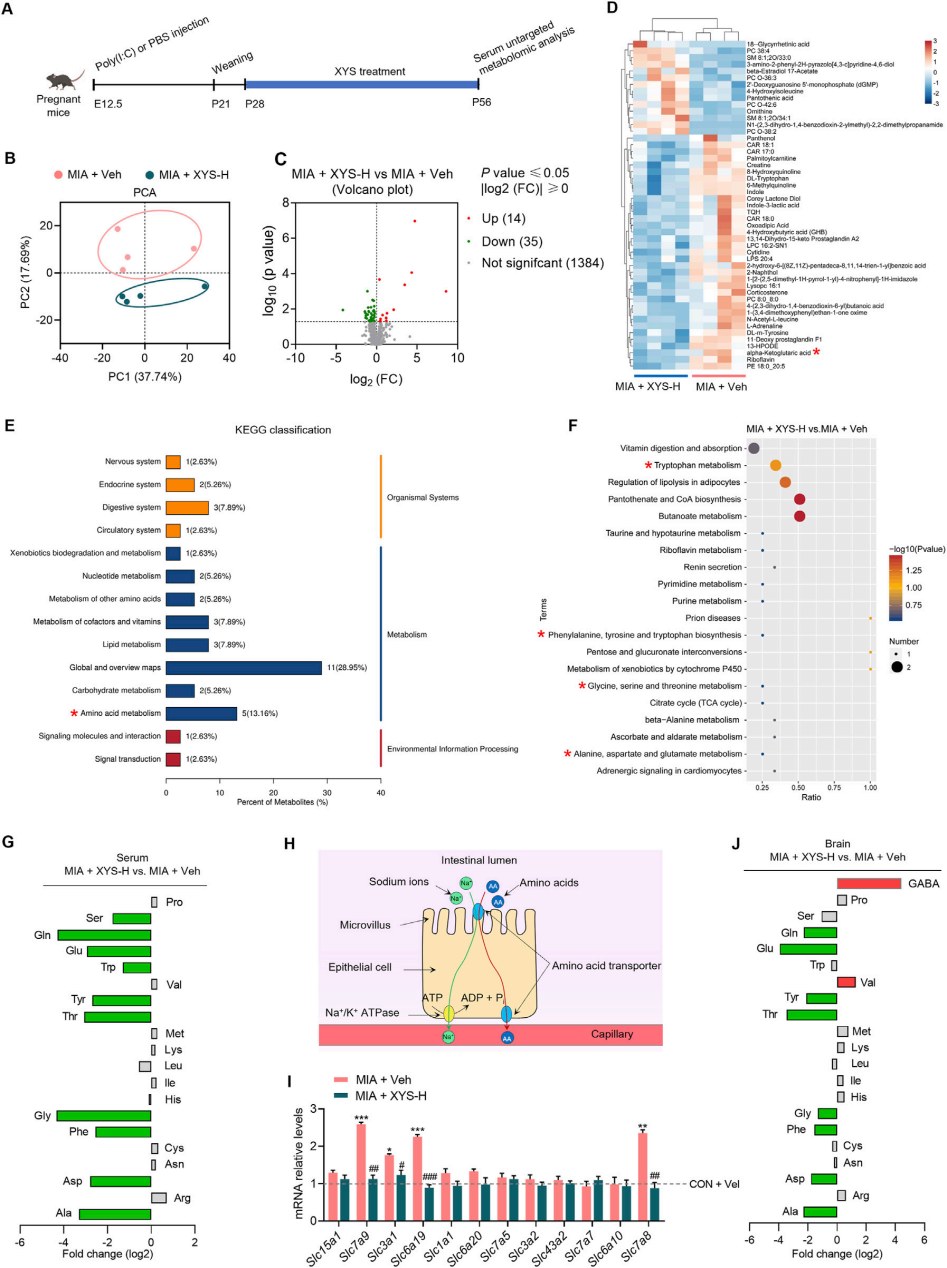
XYS alters the amino acid profile in the serum and brain of MIA offspring. **(A)** The experimental timeline for XYS treatment and serum untargeted metabolomic analysis. **(B)** PCA of the serum untargeted metabolomic data from MIA + Veh and MIA + XYS-H mice (n = 4 mice from different dams for each group). **(C)** Volcano plot of serum metabolites from MIA + Veh and MIA + XYS-H mice (n = 4 mice from different dams for each group). **(D)** Heat maps for clustering of serum differential metabolites between MIA + Veh and MIA + XYS-H mice (n = 4 mice from different dams for each group). **(E)** KEGG classification of the serum differential metabolites between MIA + Veh and MIA + XYS-H mice. **(F)** KEGG enrichment analysis of the serum differential metabolites between MIA + Veh and MIA + XYS-H mice. **(G)** Amino acid levels in MIA + XYS-H mice serum were normalized and shown as fold change (log_2_ transformed) to levels in age-matched vehicle-treated controls. The amino acids with a fold change of >1.3 and *P* value <0.05 are in red and green. **(H)** Diagram of the mechanism of intestinal absorption of amino acids. **(I)** qPCR analysis of amino acids transporters' mRNA levels in the small intestine of the test mice (n = 4 mice from different dams for each group). **(J)** The amino acid and GABA levels in the brain of MIA + XYS-H mice were normalized and shown as fold change (log_2_ transformed) to levels in age-matched vehicle-treated controls. The amino acids with a fold change of >1.3 and *P* value <0.05 are in red and green. Statistical details are provided in Supplementary Table S4.

Given the gut microbiota’s distance from the brain, we hypothesized that specific metabolites regulated by the microbiome could enter the bloodstream, influencing the positive impact of XYS on autistic behaviors in MIA mice. To support this hypothesis, we employed untargeted metabolomic profiling to pinpoint candidate microbiome-associated molecules that exhibited significant differences in abundance in the serum of both vehicle-treated and XYS-treated MIA mice at P63 (Figure 4A). PCA plot showed significant distinct metabolite profiles between XYS-H-supplemented mice and vehicle-treated controls (Figure 4B). 1433 serum metabolites were identified in the serum of mice. XYS-H supplementation significantly altered 49 metabolites, 35 metabolites were decreased, and 14 metabolites were increased (Figures 4C, D). Notably, among the differentially regulated metabolites, α-ketoglutarate (αKG), a tricarboxylic acid (TCA) cycle metabolite that regulates Gln and Glu biosynthesis (Kuhn and van Bilsen, 2022; Yu et al., 2022), was substantially downregulated in XYS-H-treated mice (Figure 4D). KEGG analysis showed that some these differential metabolites are enriched in amino acid metabolism (Figures 4E, F). Next, we used a targeted metabolomics assay to examine the serum levels of twenty standard amino acids. The results showed the levels of some amino acids (e.g., Glu, Gln, alanine, aspartate, glycine, phenylalanine, tyrosine, tryptophan, threonine, serine) were decreased in the serum of XYS-H-treated mice (Figure 4G). These observations suggest that XYS downregulates serum metabolites associated with amino acid biosynthesis.

We further investigated the mechanisms that led to decreased levels of serum amino acids in the XYS-H-treated mice. The intestinal transport of amino acids to the bloodstream is one of the primary sources of serum amino acids (Figure 4H). Using qPCR, we investigated the amino acid transporters' mRNA levels in the small intestine. Our results indicated that MIA enhanced the expression of several transporters for amino acids (*Slc7a9*, *Slc3a1*, *Slc6a19*, and *Slc7a8*) in the intestine (Figure 4I). XYS treatment normalized the mRNA levels of amino acid transporters to control levels (Figure 4I), demonstrating that XYS reduces amino acid absorption in the intestine by reducing the expression of these transporters.

Abnormalities in Gln in the brain are linked to the pathophysiology of neurological diseases and mental illnesses. (Lee et al., 2013; Andersen et al., 2021a; Sandhu et al., 2021; Yu et al., 2022; Wang P. et al., 2023). Gln can cross the blood-brain barrier (BBB) and is the precursor for synthesizing Glu and GABA, two primary neurotransmitters in the brain, playing crucial roles in neural signaling and overall brain function (Bak et al., 2006). Thus, we measured the amino acids and GABA levels in the mPFC of mice by targeted metabolomics assay. We found that XYS treatment decreased the Gln and Glu levels in the mPFC of MIA offspring compared with vehicle treatment (Figure 4J). Surprisingly, The GABA levels were increased in the mPFC of XYS-treated mice compared with vehicle-treated controls (Figure 4J). These results suggested that XYS treatment alters the Glu/GABA balance with a decreased Glu/GABA ratio.

These results suggested that XYS treatment alters the Glu/GABA balance with a decreased Glu/GABA ratio. It has been previously reported that MIA alters E-I balance in the hippocampus and cortex of the offspring (Okamoto et al., 2018; Fernandez et al., 2019; Griego et al., 2025). A reduced Glu/GABA ratio in the prefrontal cortex, combined with alterations in other metabolites, may underlie social behavior deficits observed in Cntnap^2−/−^ mice (Park et al., 2022). Our results support the idea that XYS improves ASD-like behaviors by restoring the Glu/GABA ratio in MIA mice.

### 3.5 B. uniformis decreases intestinal amino acid transporter levels and restores Glu/GABA balance in MIA offspring

**FIGURE 5:**
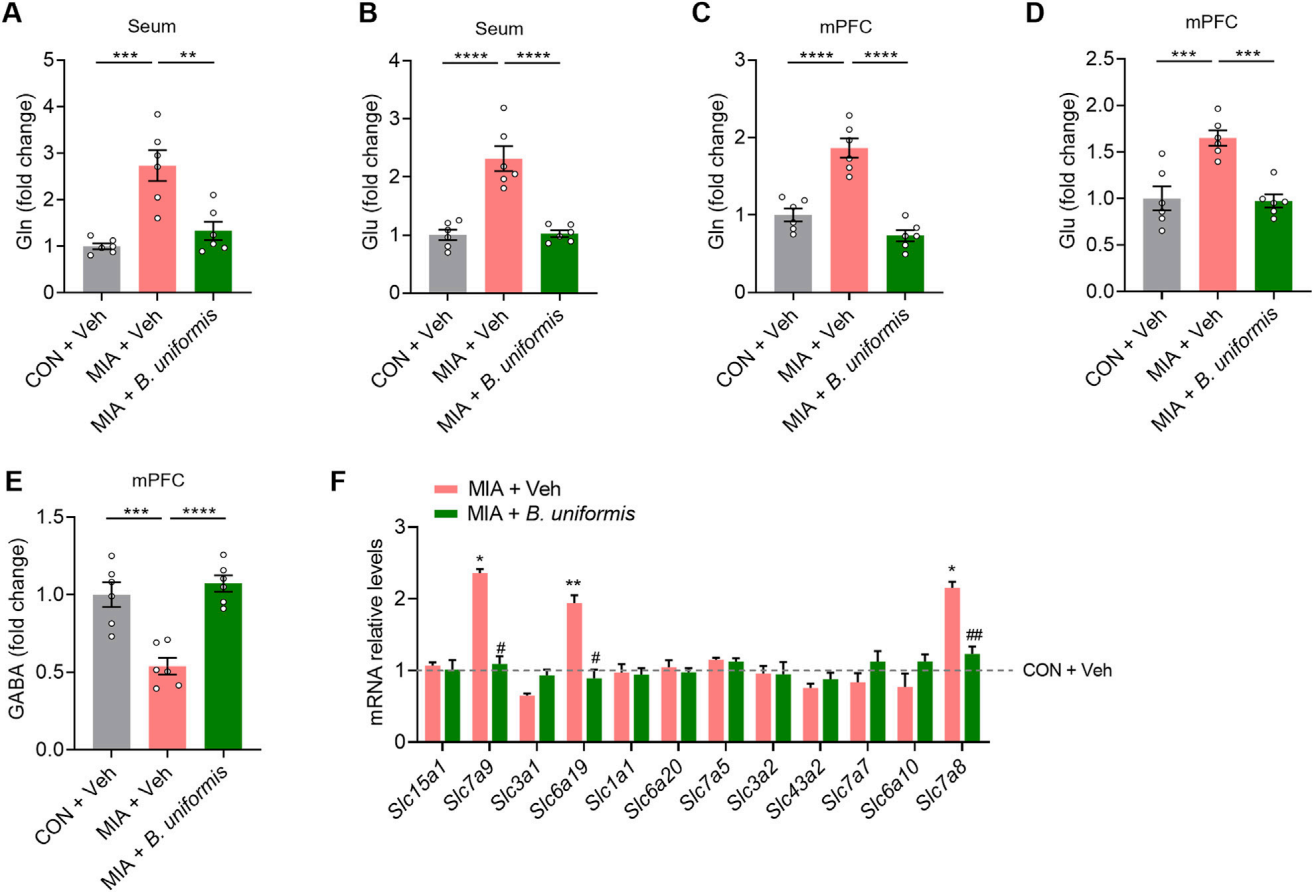
*Bacteroides uniformis* restores Glu/GABA balance in MIA offspring. **(A, B)** The levels of Gln and Glu in the serum. **(C–E)** Gln, Glu, and GABA levels in the mPFC. **(F)** qPCR analysis of amino acids transporters' mRNA levels in the small intestine of the test mice (n = 5 mice from different dams for each group). Graphs are mean ± SEM. Statistical details are provided in Supplementary Table S4.

We next investigated how *B. uniformis* affects the Gln, Glu, and GABA levels in the mPFC of the MIA mice. Treatment with *B. uniformis* restored the levels of Gln and Glu in both the serum and the mPFC to standard ranges (Figures 5A–D). Additionally, *B. uniformis* increased GABA levels and restored the Glu/GABA balance in the mPFC of MIA mice (Figure 5E). These findings suggest that *B. uniformis* corrects Glu/GABA balance in MIA mice.

To investigate the effect of *B. uniformis* treatment on the mRNA levels of intestinal amino acid transporters in MIA mice, we conducted qRNA analysis of small intestinal tissue. Our results showed that treatment with *B. uniformis* reduced the mRNA levels of several intestinal amino acid transporters (*Slc6a19*, *Slc7a8*, and *Slc7a15*) in the MIA mice (Figure 5F). The results indicate that *B. uniformis* significantly reduces the levels of Gln and Glu in serum by inhibiting the expression of intestinal amino acid transporters. These findings suggest that XYS is beneficial in diminishing intestinal amino acid transport in offspring affected by MIA through regulating *Bacteroides*.

### 3.6 XYS promotes GABAergic signals and prevents MIA-induced increase in astrocytes within the mPFC of MIA offspring

**FIGURE 6:**
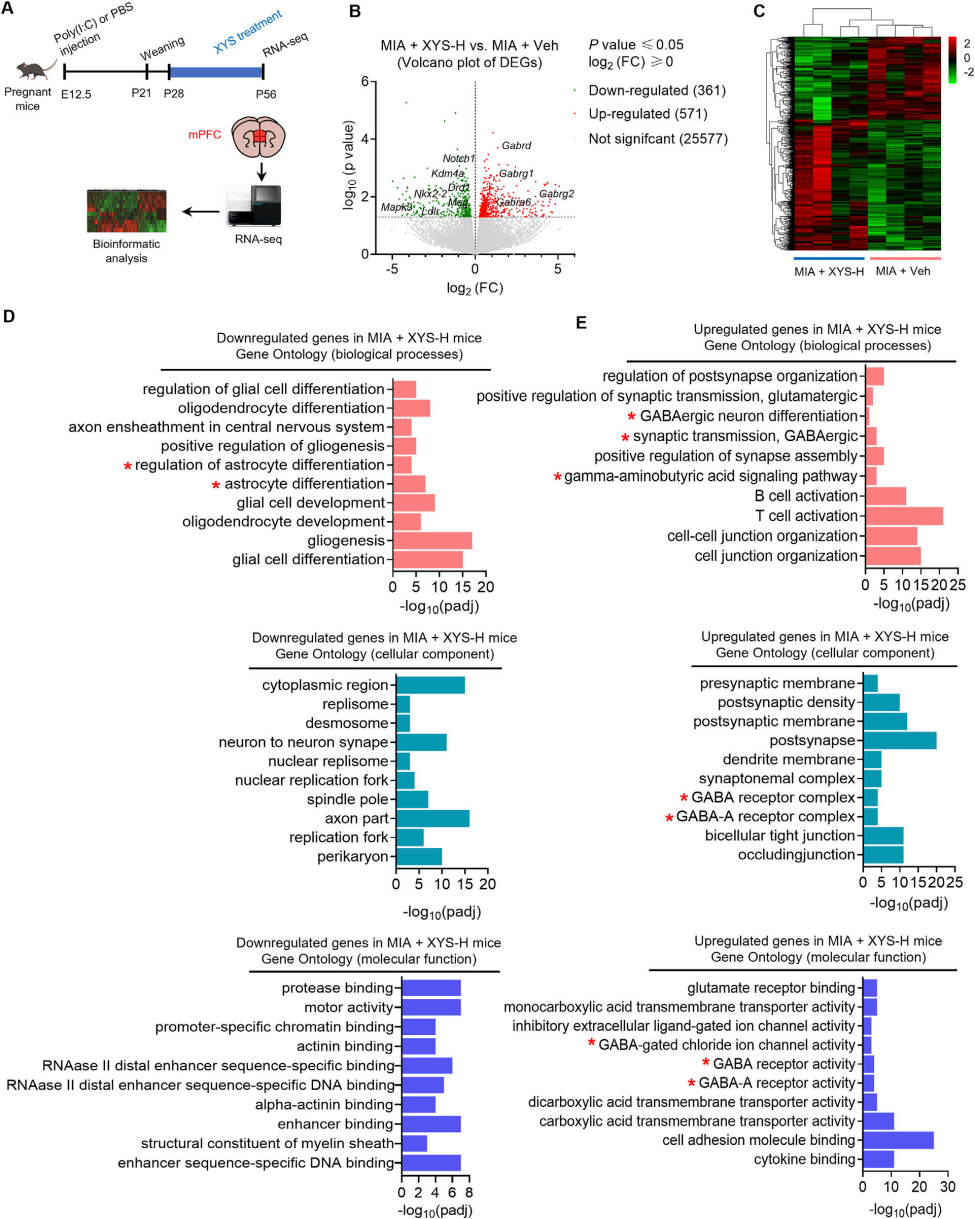
XYS upregulates GABAergic signaling-related genes and downregulates astrocyte differentiation-related genes in mPFC of MIA offspring. **(A)** Strategies for RNA-seq in the mPFC. **(B)** Heat maps for mPFC differentially expressed genes clustering between MIA + Veh and MIA + XYS-H mice (n = 4 mice from different dams for each group). **(C)** Volcano plot of mPFC differentially expressed genes between MIA + Veh and MIA + XYS-H mice (n = 4 mice from different dams for each group). **(D, E)** GO Enrichment analysis of the differentially expressed genes between MIA + Veh and MIA + XYS-H mice. GO terms for biological processes (top); GO terms for cellular components (middle); GO terms for molecular functions (bottom).

Amino acids have been well-known participants in neurotransmission. Moreover, evidence has recently been accumulating that amino acids regulate gene expression and the protein phosphorylation cascade (Jousse et al., 2004; Bruhat et al., 2009). We then explored transcriptome alterations in mPFC in XYS-treated MIA mice to understand better how amino acids are implicated in the alleviation effects of XYS on ASD-like phenotypes in MIA offspring using RNA-seq (Figure 6A). In comparison to vehicle-treated controls, we detected 361 downregulated genes and 571 upregulated genes in the mPFC of XYS-treated MIA offspring (Figures 6B, C). Gene ontology (GO) analysis showed that The downregulated genes are mainly enriched in glial cell differentiation, especially the astrocyte differentiation and oligodendrocyte differentiation (Figure 6D). Furthermore, we found that the GABAergic signaling pathway-related genes were upregulated genes in XYS-treated MIA offspring (Figure 6E). The increase of the mRNAs levels of GABA_A_ receptors (*Gabra6* and *Gabrd*) in the mPFC of XYS-treated MIA offspring was confirmed by qPCR (Supplementary Figure S4).

At the cellular level, Glu and GABA are primarily taken up by astrocytes at the synapse (Bak et al., 2006; Schousboe et al., 2014). The communication between neurons and astrocytes is a classic example of cell-cell signaling that involves the Gln and Glu/GABA cycle (Schousboe et al., 2013; Schousboe et al., 2014). Astrocytes play a crucial role in supporting neuronal metabolism and preventing the extracellular accumulation of neurotransmitters, thereby reducing the risk of excitotoxicity (Schousboe et al., 2014; Andersen et al., 2021b). The activity of astrocytes is partially indicated by the synthesis of Glu (Schousboe et al., 2014). Therefore, extracellular Glu/GABA ratios reflect the E/I balance in specific brain regions (Andersen et al., 2021b).

**FIGURE 7:**
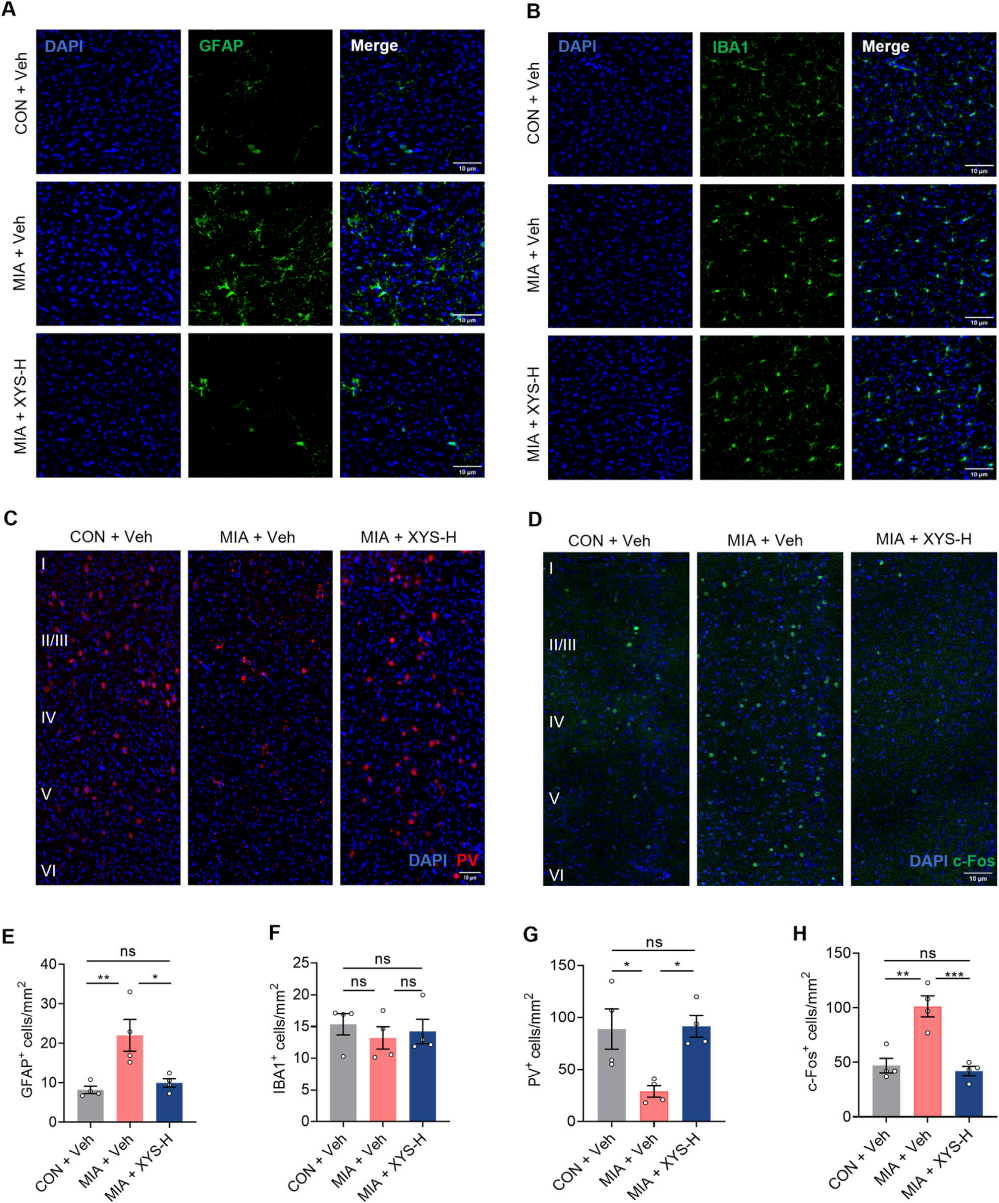
XYS reduces hyperactivation in the mPFC of MIA offspring. **(A)** Representative images illustrating GFAP (green) expression in the mPFC. Scale bars, 10 μm. **(B)** Representative images illustrating Iba1 (green) expression in the mPFC. Scale bars, 10 μm. **(C)** Representative images illustrating PV (green) expression in the mPFC. Scale bars, 10 μm. **(D)** Representative images illustrating c-Fos (green) expression in the mPFC. Scale bars, 10 μm. **(E)** Quantification of GFAP-expressing cells in the mPFC. **(F)** Quantification of Iba1-expressing cells in the mPFC. **(G)** Quantification of PV-expressing cells in the mPFC. **(H)** Quantification of c-Fos-expressing cells in the mPFC. n = 4 mice from different dams for each group. Graphs are mean ± SEM. Statistical details are provided in Supplementary Table S4.

Our RNA-seq data showed that XYS treatment inhibits the differentiation of astrocytes. Thus, we invested the number of astrocytes in the mPFC using immunohistochemical analysis. Our results showed that the astrocytes (GFAP^+^ cells) were reduced in XYS-treated MIA offspring (Figures 7A, E). XYS did not influence the number of microglia cells (Iba1^+^) in the mPFC (Figures 7B, F). Together, these results suggest that XYS, at least in part, reduces the levels of Glu in the mPFC by preventing the MIA-induced increase in astrocytes.

Additionally, Our RNA sequencing data indicated that XYS treatment enhances the differentiation of GABAergic interneurons (Figure 6E). Previous studies have shown that maternal inflammation can disrupt the functioning of cortical GABAergic interneurons, which may contribute to the abnormal behaviors commonly observed in neurodevelopmental disorders among offspring exposed to maternal inflammation (Canetta et al., 2016; Shin Yim et al., 2017; Vasistha et al., 2020; Zhang et al., 2023). We conducted an immunohistochemical analysis to assess the number of interneurons in the mPFC. Our data demonstrate that MIA exposure in offspring leads to reduced parvalbumin (PV) expression in the mPFC, reflecting a selective loss of a subclass of these fast-spiking interneurons (Figures 7C, G). Notably, treatment with XYS significantly increased the number of PV^+^ interneurons in the mPFC of the MIA offspring (Figures 7C, G).

Changes in the number of astrocytes and PV^+^ interneurons may lead to alterations in neural activity within the brain. Therefore, we investigated whether the behavioral improvement induced by XYS in MIA offspring is linked to changes in neural activity in the mPFC. Our findings revealed that MIA offspring showed increased c-Fos^+^ (a marker for neuronal activation) cells in the mPFC (Figures 7D, H). Interestingly, XYS treatment decreased the number of c-Fos^+^ cells in the mPFC to levels similar to those of the control offspring (Figures 7D, H). In summary, the behavioral improvement induced by XYS in MIA offspring was associated with a reduction in neural activity, achieved by promoting GABAergic signals and preventing MIA-induced increase in astrocytes in the mPFC of offspring.

## 4 Discussion

**FIGURE 8:**
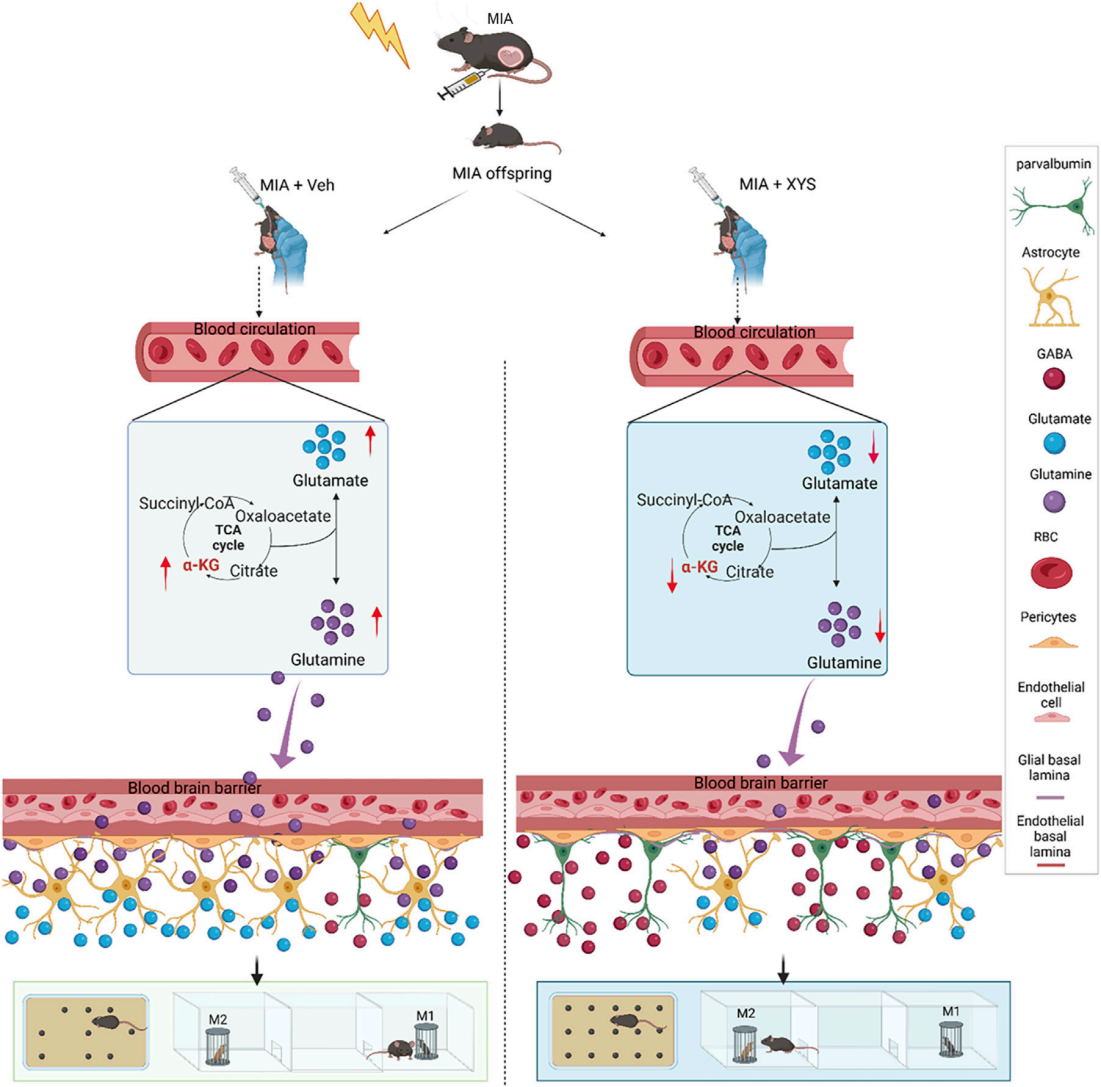
Working model: XYS mitigates neurobehavioral abnormalities in MIA mice by regulating the gut microbiome, remarkably increasing *Bacteroides*, and modulating serum amino acids and brain neurotransmitters. It restores the excitation/inhibition balance, inhibits astrocyte differentiation, and enhances GABAergic signaling, alleviating autism-like behaviors.

The present study investigates the therapeutic potential of XYS, a traditional Chinese medicine, in ameliorating neurobehavioral abnormalities in a Poly (I:C)-induced MIA mouse model associated with ASD. Our findings contribute to understanding the gut-microbiome-brain axis and provide insights into a novel therapeutic approach for ASD. The comprehensive modulation of the gut microbiome, systemic metabolites, and neural pathways by XYS highlights the multi-target, multi-therapeutic potential of TCM. Our results demonstrate that XYS treatment significantly improves MIA-associated behavioral abnormalities by reducing repetitive behaviors and enhancing social interaction in the offspring. This is in line with XYS’s historical use in treating stress-related disorders and mood disorders, suggesting a broader application in ASD. We elucidated the role and molecular mechanism of gut microbiota and metabolites in the improvement of autistic-like behaviors by XYS that gut microbiota may mediate the XYS’s alleviation effects by modulating serum levels of α-KG and amino acids, which controls Gln availability and maintains glutamatergic and GABAergic function in the mPFC (Figure 8).

One of the most intriguing findings of our study is the observation that treatment with XYS in post-weaning mice can reverse the behavioral and neurodevelopmental effects induced by MIA during the prenatal period. This finding suggests that the impact of early-life events, such as MIA, on neurodevelopmental trajectories may not be irreversible and can be modulated by interventions later in life (Hsiao et al., 2013; Reed et al., 2020). The brain retains a significant degree of plasticity even after the critical periods of early development (Hübener and Bonhoeffer, 2014; Marzola et al., 2023). This plasticity allows for the reorganization and remodeling of neural circuits in response to environmental stimuli and interventions (Hübener and Bonhoeffer, 2014; Marzola et al., 2023). Our findings suggest that XYS may harness this plasticity to reverse the adverse effects of MIA on neural development. For example, XYS may modulate synaptic plasticity, neurotransmitter levels, and neuroinflammatory processes, thereby restoring normal neural function.

The gut microbiome plays a crucial role in modulating brain function and behavior through the gut-brain axis (Ahrens et al., 2024; Qu et al., 2024). Our study demonstrates that XYS treatment alters the gut microbiome composition, which in turn may influence neural development and behavior. This suggests that the gut microbiome could serve as a therapeutic target for reversing the effects of early-life insults such as MIA (Loh et al., 2024). The mechanisms underlying this interaction may involve the production of neuroactive metabolites, modulation of immune responses, and direct effects on neural signaling pathways. Future research should aim to elucidate the specific mechanisms by which XYS reverses the effects of MIA. Additionally, studies should explore the potential therapeutic applications of XYS and similar interventions in clinical settings, particularly in individuals with neurodevelopmental disorders.

The gut microbiome’s pivotal role in modulating host behavior and brain function has been increasingly recognized (Bravo et al., 2011; Cryan and Dinan, 2012; Foster and McVey Neufeld, 2013). The modulation of the gut microbiome by XYS is a crucial finding of our study. Our findings indicate that XYS significantly alters the gut microbiome composition, remarkably increasing the abundance of *Bacteroides*. This alteration is associated with improvements in behavioral phenotypes reminiscent of ASD, suggesting a gut microbiome-brain axis at play. Restoring the Glu/GABA balance in the brain by supplementation of *B. uniformis* further supports the microbiome’s regulatory influence on neural function.

The untargeted metabolomic profiling revealed that XYS treatment led to significant alterations in serum metabolites, particularly those related to amino acid metabolism, which are crucial for neurotransmitter synthesis and neural function (Novarino et al., 2012; Tarlungeanu et al., 2016; Yu et al., 2022; Knaus et al., 2023). The decrease in serum amino acids, particularly Gln, may contribute to normalizing neurotransmitter levels and the E/I balance (Yu et al., 2022; Wang P. et al., 2023). This finding aligns with the emerging view that peripheral metabolic changes can influence central nervous system function (Zeng et al., 2022), a concept that TCM has long embraced through the lens of holistic health. Our study also explored the molecular mechanisms by which XYS exerts its effects. The molecular mechanisms underlying XYS’s therapeutic effects involve the modulation of intestinal amino acid transporters, astrocyte differentiation, and GABAergic signaling. These mechanisms converge on regulating neural activity and synaptic function, indicative of XYS’s potential to promote neural plasticity. The reduction in GFAP expression, and the normalization of c-Fos expression, a marker of neuronal activity, suggest that XYS may attenuate excitatory signaling and enhance inhibitory tone, thereby rebalancing neural circuitry.

In our study, we observed a reduction in PV expression in the mPFC of MIA-exposed offspring, which we interpreted as indicative of a potential loss or dysfunction of a specific class of fast-spiking interneurons. This observation aligns with previous research demonstrating that PV interneurons play a crucial role in maintaining the excitation/inhibition (E/I) balance in neural circuits (Ferguson and Gao, 2018; Zhang et al., 2025). However, we acknowledge that our study did not directly test markers for other interneuron populations, such as somatostatin (SST) or vasoactive intestinal peptide (VIP) interneurons. Therefore, we cannot definitively conclude the specificity of the observed effects to PV interneurons alone.

The results of this study have important implications for the treatment of ASD. By targeting the gut microbiome and modulating systemic metabolites, XYS may offer a non-invasive and potentially more tolerable therapeutic option than conventional pharmacological interventions. Furthermore, identifying *B. uniformis* as a key player in mediating the effects of XYS opens up avenues for targeted probiotic therapies. TCM’s multi-target approach contrasts the often single-target focus of Western medicine (Yu et al., 2025). The pleiotropic effects of XYS, affecting the gut microbiome, peripheral metabolism, and central nervous system function, exemplify the integrative nature of TCM. This holistic strategy may offer advantages in treating disorders with complex etiologies like ASD, where multiple systems and pathways are implicated. Integrating TCM with modern medicine is a burgeoning field, with XYS serving as a model compound. The mechanistic insights from our study could facilitate the development of biomarkers for TCM efficacy and help bridge the gap between traditional practices and evidence-based medicine.

Moreover, identifying specific microbial strains like *B. uniformis* opens avenues for precision medicine, where personalized probiotic therapies could be tailored to individual microbiome profiles. Recent advances in neurodevelopmental research have highlighted the importance of early life factors, such as MIA, in shaping long-term brain health (Kim et al., 2017; Shin Yim et al., 2017; Reed et al., 2020). Our study contributes to this field by demonstrating that early interventions with TCM, like XYS, can have lasting effects on neurobehavioral outcomes. The modulation of the gut microbiome by XYS may represent a novel preventive or therapeutic strategy for ASD, warranting further exploration.

While our study provides valuable insights into the potential effects of XYS on neurodevelopmental outcomes in the context of MIA, we recognize several limitations inherent in our approach and the broader field of traditional medicine research. Traditional medicine, including the use of herbal formulas like XYS, has been practiced for centuries and often demonstrates therapeutic efficacy. However, the mechanisms of action underlying these effects are frequently not well understood. Our study, while suggesting that XYS may modulate the gut microbiome and influence neurodevelopmental outcomes, does not provide a comprehensive mechanistic explanation. Future research should focus on elucidating the specific pathways and molecular mechanisms through which XYS exerts its effects. This includes investigating the role of individual herbal components, their interactions, and their impact on various biological systems. The potential side effects of traditional medicine are often understudied. While XYS has been used in clinical practice for many years, systematic investigations into its long-term effects, potential interactions with other medications, and impact on different patient populations are still needed. Ensuring the safety of traditional medicine formulations is paramount, particularly given their widespread use and potential for integration into modern healthcare. We believe that addressing these limitations will enhance the scientific rigor and clinical applicability of traditional medicine research.

In summary, our study provides a comprehensive view of the therapeutic potential of XYS in a preclinical model of ASD. The multi-target, multi-therapeutic approach of XYS, grounded in the principles of TCM, offers a promising avenue for developing novel treatments for ASD. By integrating the insights from our study with the latest research frontiers, we can pave the way for a more holistic and personalized approach to managing these complex disorders.

## Data Availability

The 16S rRNA and transcriptomic sequencing datasets are available from the NCBI SRA database with the accession numbers PRJNA1120764 and PRJNA1126137. Other data supporting this study’s findings are available upon reasonable request from the corresponding authors. More information on data analysis is available in the Supplementary Material and Methods section.

## Abbreviations

ADHD, Attention deficit hyperactivity disorder; ASD, Autism spectrum disorder; DEG, Differential expression gene; MIA, Maternal immune activation; ELISA, Enzyme-linked immunosorbent assays; EPM, Elevated Plus Maze; GO, Gene Ontology; KEGG, Kyoto Encyclopedia of Genes and Genomes; MIA, Maternal immune activation; NDDsNeurodevelopmental disorders; OTUs, Operational taxonomic units; PCA, Principal component analysis; PCoA, Principal coordinate analysis; PFA, Paraformaldehyde; PPI, Protein-protein interaction; PV, Parvalbumin; RNA-seq, RNA sequencing; SPF, Specific pathogen-free; TCM, Traditional Chinese medicine; XYS, Xiaoyao San.
