# Supplementary material for: Xiaoyao San ameliorates maternal inflammation-induced neurobehavioral deficits by modulating the microbiota-gut-brain axis in offspring
Source: Front Pharmacol. 2025 May 19;16:1563496. doi: 10.3389/fphar.2025.1563496 (PMC12127538; doi:10.3389/fphar.2025.1563496)
Supplement: Supplementary file 1 [file Table1.docx]

**Supplementary Information**

**Supplementary Table 1.** Primers used in this study

| Gene ID | Amplification product size (bp) | Primers | Sequence (5’→3’) | Length | Tm (℃) | location |
| --- | --- | --- | --- | --- | --- | --- |
| 56643 | 173 | SLC15A1-F | CCGGCACACCCTTCTAGTG | 19 | 62.0 | 1377-1395 |
|  |  | SLC15A1-R | TGGCGTTGTGACTGGTGAC | 19 | 62.5 | 1549-1531 |
| 30962 | 172 | SLC7A9-F | GAGGAGACGGAGAGAGGATGA | 21 | 61.6 | 18-38 |
|  |  | SLC7A9-R | CCCCACGGATTCTGTGTTG | 19 | 60.4 | 189-171 |
| 74338 | 105 | SLC6A19-F | CAGGTGCTCAGGTCTTCTACT | 21 | 60.6 | 812-832 |
|  |  | SLC6A19-R | CGATCACAGAATCCATCTCACAA | 23 | 60.1 | 916-894 |
| 20510 | 176 | SLC1A1-F | CTTCCTACGGAATCACTGGCT | 21 | 61.3 | 39-59 |
|  |  | SLC1A1-R | CGATCAGCGGCAAAATGACC | 20 | 62.0 | 214-195 |
| 20538 | 227 | SLC6A20-F | CCTGCAAAACCGCCGATCTA | 20 | 62.6 | 80-99 |
|  |  | SLC6A20-R | GAGGAACAGCGTGTATGGAATC | 22 | 60.7 | 306-285 |
| 20539 | 221 | SLC7A5-F | ATATCACGCTGCTCAACGGTG | 21 | 62.8 | 149-169 |
|  |  | SLC7A5-R | CTCCAGCATGTAGGCGTAGTC | 21 | 62.0 | 369-349 |
| 17254 | 183 | SLC3A2-F | TGATGAATGCACCCTTGTACTTG | 23 | 60.8 | 857-879 |
|  |  | SLC3A2-R | GCTCCCCAGTGAAAGTGGA | 19 | 61.2 | 1039-1021 |
| 215113 | 133 | SLC43A2-F | TGCACCGCTGTGTTGGAAA | 19 | 62.7 | 49-67 |
|  |  | SLC43A2-R | CCGTGCTGTTAGTGACATTCTC | 22 | 60.9 | 181-160 |
| 20540 | 177 | SLC7A7-F | CACCACCAAGTATGAAGTGGC | 21 | 60.9 | 9-29 |
|  |  | SLC7A7-R | CCCTTAGGGGAGACAAAGATGC | 22 | 62.1 | 185-164 |
| 72472 | 147 | SLC16A10-F | GAGGTGGAGCTGACGAGGT | 19 | 63 | 139-157 |
|  |  | SLC16A10-R | CATGGACACGAAGAGCACCC | 20 | 62.8 | 285-266 |
| 50934 | 219 | SLC7A8-F | TGTGACTGAGGAACTTGTGGA | 21 | 60.7 | 756-776 |
|  |  | SLC7A8-R | GTGGACAGGGCAACAGAAATG | 21 | 61.5 | 974-954 |
| 14405 | 224 | Gabrg1-F | GCGTGAGACCCACAGTGATT | 20 | 62.2 | 251-270 |
|  |  | Gabrg1-R | TGCATCCGATTTTCTTGAGTTCC | 23 | 61.1 | 474-452 |
| 14406 | 85 | Gabrg2-F | ATGAGTTCGCCAAATACATGGAG | 23 | 60.7 | 1-23 |
|  |  | Gabrg2-R | GGAGCAGAATCCACAGCGT | 19 | 62 | 85-67 |
| 14399 | 98 | Gabra6-F | TGCCCAAGCTCAACTTGAAGA | 21 | 61.9 | 48-68 |
|  |  | Gabra6-R | GCCGTAGACGGTTGTCATAGC | 21 | 62.8 | 145-125 |
| 14403 | 190 | Gabrd-F | CCAGCATTGACCATATCTCAGAG | 23 | 60.2 | 218-240 |
|  |  | Gabrd-R | TCATGGAACCAGGCAGATTTG | 21 | 60.3 | 407-387 |
| 14433 | 123 | Gapdh-F | AGGTCGGTGTGAACGGATTTG | 21 | 62.6 | 8-28 |
|  |  | Gapdh-R | TGTAGACCATGTAGTTGAGGTCA | 23 | 60.2 | 130-108 |

**Supplementary Table 2.** Primary antibodies used in this study.

| Primary antibodies | Source | Product code | Source/Isotype |
| --- | --- | --- | --- |
| Phospho-c-Fos (Ser32) (D82C12) XP^®^ Rabbit mAb | Cell Signaling | 5348S | Rabbit IgG |
| Monoclonal anti-parvin mouse antibody | Sigma-Aldrich | P3088 | Mouse  IgG |
| Anti-GFAP antibody | Abcam | Ab4674 | Chicken IgY |
| Iba1 Rabbit Polyclonal Antibody | Beyotime | AF7143 | Rabbit IgG |

**Supplementary Table 3.** Second antibodies used in this study.

| Second antibody | Source | Product code | Host | Isotype | Target species |
| --- | --- | --- | --- | --- | --- |
| Goat Anti-Mouse IgG H&L (Alexa Fluor® 555) | Abcam | ab150118 | Goat | IgG | Mouse |
| Goat Anti-Chicken IgY H&L (Alexa Fluor® 405) | Abcam | ab175674 | Goat | IgG | Chicken |
| Donkey anti-Rabbit IgG(H+L) Highly Cross-Adsorbed Secondary antibody, Alexa Fluor^TM^ 488 | Thermo Fisher Scienific | A-21206 | Donkey | IgG | Rabbit |

**
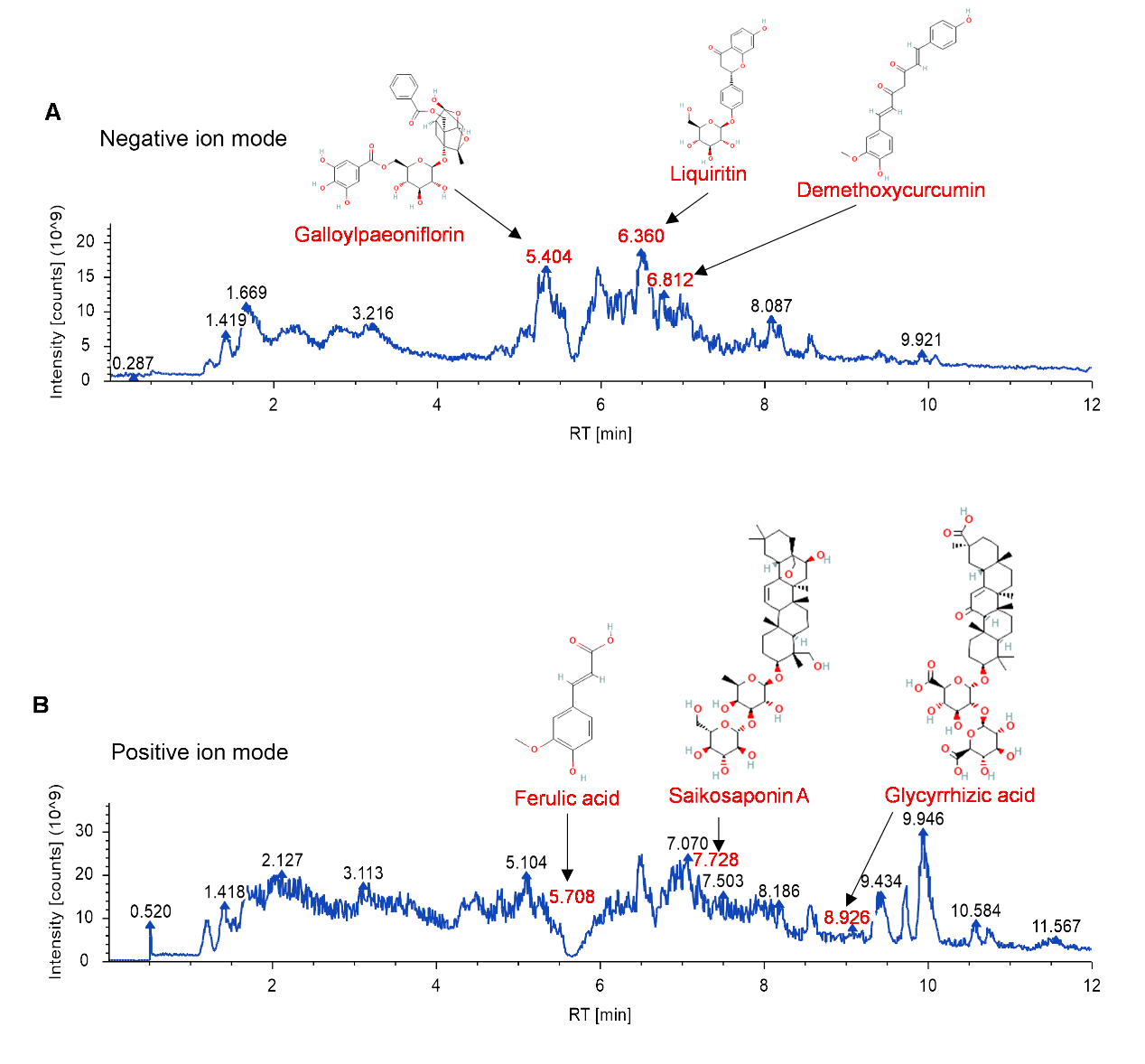
Supplementary Figures**

**
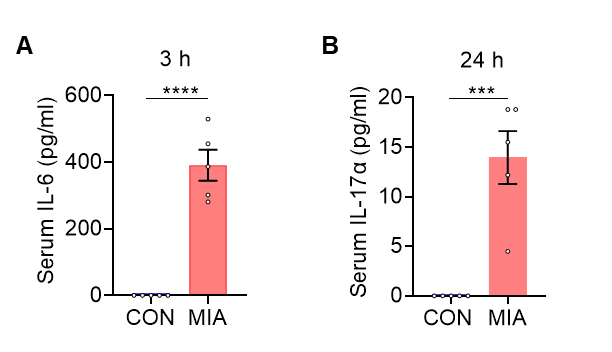
Supplementary Figure 1.** Chemical profile of XYS. (A) Chemical profile analysis of XYS by negative ion mode; (B) Chemical profile analysis of XYS by positive ion mode.

**Supplementary Figure 2.** Poly (I:C) induces upregulation of IL-6 and IL-17α in maternal plasma. (**A**) Upregulation of IL-6 protein in maternal plasma 3 h after injection of poly (I:C). (**B**) Upregulation of IL-17α protein in maternal plasma 24 h after injection of poly I:C. n = 5 mice for each group. Data were shown as mean ± SEM. ****P* < 0.001, *****P* < 0.0001. Statistical differences were determined by two-tailed unpaired Student's *t*-test (**A**, **B**). Statistical details are provided in Supplementary Table 5.


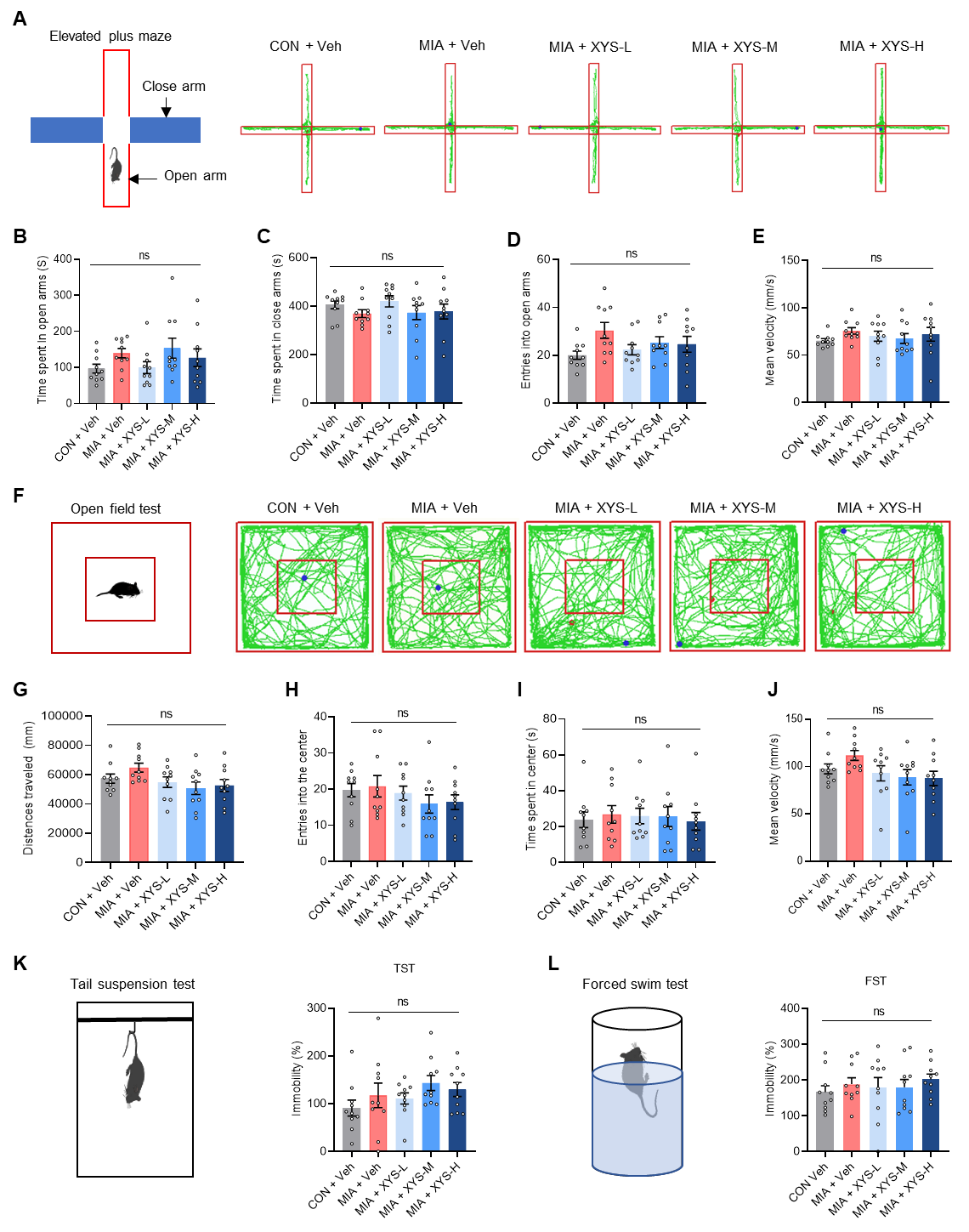
**Supplementary Figure 3.** (**A**) Representative traces in the elevated plus maze test. (**B**) Time spent in the open arms in elevated plus maze test (n = 10 for each group). (**C**) Time spent in the closed arms in elevated plus maze test (n = 10 for each group). (**D**) Number of entries in the open arms in forced swim test (n = 10 for each group). (**E**) The moving speed of test mice in elevated plus maze test (n = 10 for each group). (**F**) Representative traces in open field test. (**G**) Distance travelled in the center in open field test (n = 10 for each group). (**H**) Number of entries in the center in open field test (n = 10 for each group). (**I**) Time spent in the center in open field test (n = 10 for each group). (**J**) The moving speed of test mice in open field test (n = 10 for each group). (D) Time of immobility in forced swim test (n = 10 for each group). (**K**) Time of immobility in tail suspended test (n = 10 for each group). (**L**) Time of immobility in forced swim test (n = 10 for each group). Data were shown as mean ± SEM. ns, no significant difference. Statistical differences were determined by one-way ANOVA with Tukey's multiple-comparison test (**B**-**E**, **G**-**J**, **K**, **L**). Statistical details are provided in Supplementary Table 5.

**
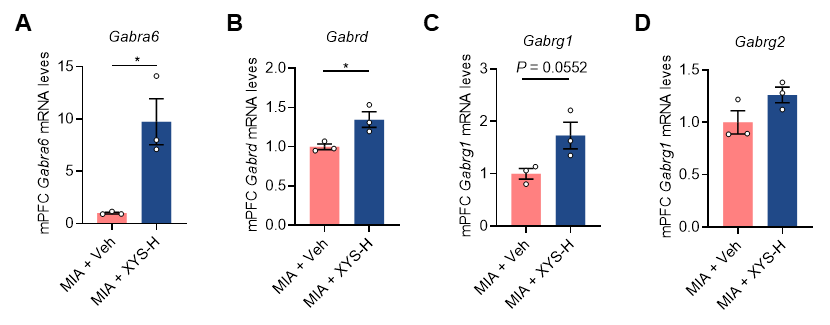
**

**Supplementary Figure 4.** XYS treatment upregulates GABA_A_ receptors-related genes. (**A**-**D**) The relative mRNA levels of *Gabra6*, *Gabrd*, *Gabrg1* and *Gabrg2* in the mPFC of vehicle- and XYS-treated MIA offspring (n = 3 mice from different dams for each group). Data were shown as mean ± SEM. **P* < 0.05. Statistical differences were determined by two-tailed unpaired Student's *t*-test (**A**-**D**). Statistical details are provided in Supplementary Table 5.
